# Prognostic Nutritional Index as a Predictor of Diabetic Nephropathy Progression

**DOI:** 10.3390/nu14173634

**Published:** 2022-09-02

**Authors:** Junlin Zhang, Xiang Xiao, Yucheng Wu, Jia Yang, Yutong Zou, Yuancheng Zhao, Qing Yang, Fang Liu

**Affiliations:** 1 Division of Nephrology, West China Hospital of Sichuan University, Chengdu 610041, China; 2 Department of Nephrology, The Third People’s Hospital of Chengdu, Chengdu 610014, China; 3 Laboratory of Diabetic Kidney Disease, Centre of Diabetes and Metabolism Research, West China Hospital of Sichuan University, Chengdu 610041, China; 4 Department of Nephrology, The First Affiliated Hospital of Chengdu Medical College, Chengdu 610500, China

**Keywords:** prognostic nutritional index, diabetic nephropathy, type 2 diabetes, end-stage renal disease

## Abstract

Malnutrition and immunologic derangement were not uncommon in patients with chronic kidney disease (CKD). However, the long-term effects of prognostic nutritional index (PNI), an immunonutrition indictor, on renal outcomes in patients with diabetic nephropathy (DN) and type 2 diabetes mellitus (T2DM) are unknown. In this retrospective cohort study, 475 patients with T2DM and biopsy-confirmed DN from West China Hospital between January 2010 and September 2019 were evaluated. PNI was evaluated as serum albumin (g/L) + 5 × lymphocyte count (10^9^/L). The study endpoint was defined as progression to end-stage renal disease (ESRD). The Cox regression analysis was performed to investigate the risk factors of renal failure in DN patients. A total of 321 eligible individuals were finally included in this study. The patients with higher PNI had a higher eGFR and lower proteinuria at baseline. Correlation analysis indicated PNI was positively related eGFR (r = 0.325, *p* < 0.001), and negatively correlated with proteinuria (r = −0.68, *p* < 0.001), glomerular lesion (r = −0.412, *p* < 0.001) and interstitial fibrosis and tubular atrophy (r = −0.282, *p* < 0.001). During a median follow-up of 30 months (16–50 months), the outcome event occurred in 164(51.09%) of all the patients. After multivariable adjustment, each SD (per-SD) increment of PNI at baseline was associated with a lower incidence of ESRD (hazard ratio, 0.705, 95% CI, 0.523–0.952, *p* = 0.023), while the hypoalbuminemia and anemia were not. For the prediction of ESRD, the area under curves (AUC) evaluated with time-dependent receiver operating characteristics were 0.79 at 1 year, 0.78 at 2 years, and 0.74 at 3 years, respectively, and the addition of PNI could significantly improve the predictive ability of the model incorporating traditional risk factors. In summary, PNI correlated with eGFR and glomerular injury and was an independent predictor for DN progression in patients with T2DM. Thus, it may facilitate the risk stratification of DN patients and contribute to targeted management.

## 1. Introduction

Diabetic nephropathy (DN), as one of the serious microvascular complications of diabetes, casts shadows on 30~40% of individuals with diabetes and has become the major cause of chronic kidney disease (CKD) worldwide [1,2]. The past few years have witnessed an alarming increase in DN and the consequent worrying health and socioeconomic burdens, especially in China [3,4,5]. Although multifactorial strategic interventions [6,7], such as dietary and lifestyle adjustments, and intense control of blood glucose and pressure, have been implemented, quite a few DN patients develop renal failure. The identification of new markers associated with DN progression is imminent, so as to better detect high-risk patients for early intervention.

Emerging evidence has demonstrated that DN is a metabolic disorder, where protein malnutrition, low-grade chronic inflammation, and immune modulation are intimately linked to the development and outcomes of the disease [8,9,10,11]. Prognostic nutritional index (PNI), evaluated by the lymphocyte count of the peripheral blood and serum albumin, is a biomarker that integrates nutritional status, immune state, and inflammation condition. It was initially established by the Japanese scholars Onodera et al. [12] in 1984 to assess the nutritional and immunological status of cancer patients undergoing gastrointestinal surgery. In recent years, PNI has gradually become a new predictor for the prognosis of many diseases, such as heart failure [13] and COVID-19 [14]. PNI had also been reported to be a predictor of mortality in elderly individuals with CKD [15]. However, the relationship between PNI and the prognosis of DN in T2DM patients is unclear.

In this retrospective cohort study, we aimed to determine whether PNI, as a noninvasive and immunonutrition status biomarker, predicted the incident ESRD in T2DM patients with biopsy-confirmed DN.

## 2. Methods 

### 2.1. Patients

This study was performed with 475 patients (≥18 years of age) with biopsy-confirmed pure DN from West China Hospital of Sichuan University between January 2010 and September 2019. The diagnosis of T2DM was according to the 2018 American Diabetes Association (ADA) diagnostic criteria [16] or self-reported. The DN was diagnosed based on the criteria established by the Renal Pathology Society (RPS) in 2010 [17]. The general principles for renal biopsy in this study were T2DM patients with renal injury (defined as eGFR decline or proteinuria) who lacked absolute contraindications, especially T2DM patients without diabetic retinopathy, or T2DM patients with short diabetic duration (<5 y). The study outcome was progression to end-stage renal disease (ESRD) or renal failure, which was defined as an eGFR < 15 mL/min/1.73 m^2^ or initiation of long-term dialysis or kidney transplantation.

**Figure 1:**
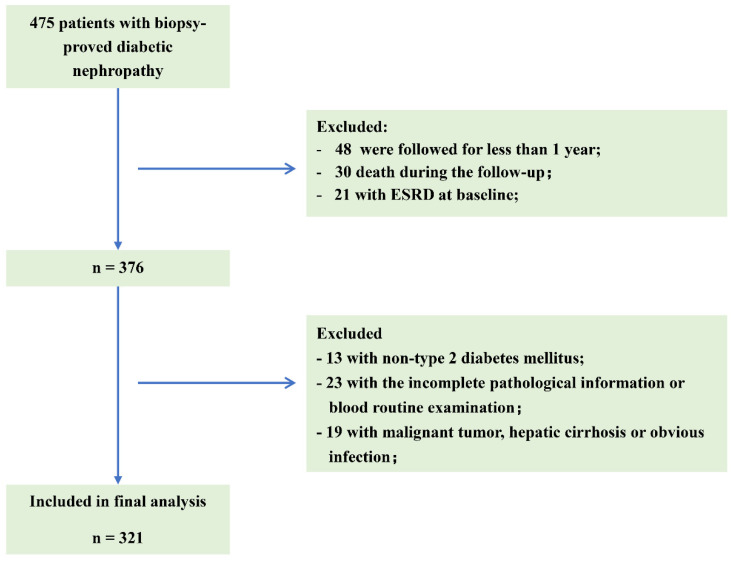
Flowchart of included patients in this study. ESRD: end-stage renal disease.

Patients with follow-up time > 1 year were eligible. Additionally, the patients who progressed to renal failure within 1 year were also included. The exclusion criteria were as follows (Figure 1): (1) patients with follow-up time < 1 year; (2) deaths that occurred during the follow-up period; (3) patients with ESRD at baseline; (4) patients with type 1 diabetes; (5) patients with incomplete pathological information or blood routine examination; (6) patients with malignant tumor, hepatic cirrhosis or active infection. 

The protocol of this research was approved by the ethics committee of West China Hospital and written informed consent were obtained from all the patient at baseline.

### 2.2. Data Collection

Clinical information of the patients was collected at baseline from the hospital information system, including (1) demographic data, such as age, gender, and ethics, etc., (2) medical history, such as hypertension, diabetes, and coronary heart disease, etc., (3) examination data including height, weight, and systolic/diastolic blood pressure, etc., (4) laboratory data including serum creatinine, blood urea nitrogen, serum lipid, uric acid, HbA1c, fasting blood sugar, blood routine, proteinuria, and pathological information, etc. The eGFR was calculated with the formula of Chronic Kidney Disease Epidemiology Collaboration [18]. Pathological lesions were routinely evaluated using light and electron microscopy by two renal pathologists based on the 2010 RPS criteria.

PNI value was evaluated as serum albumin (g/L) + 5 × lymphocyte count (10^9^/L). The anemia was defined as hemoglobin levels < 130 g/L for males and <120 g/L for females. Hypoalbuminemia was defined as serum albumin < 35 g/L. The early onset of T2DM was defined as the age of T2DM diagnosis being less than 40 years old.

### 2.3. Statistical Analysis

All data were analyzed using SPSS 26.0 statistical software (SPSS, Chicago, IL, USA) and R version 4.1.3 (http://www.rproject.org, accessed on 20 April 2022). Variables were presented as the mean ± standard, the medians with interquartile ranges (25th and 75th percentiles) or counts and percentages. The two-tailed one-way ANOVA or Kruskal–Wall was used to analyze the differences among groups. Chi-squared test was used to compare the categorical variables. Correlations between variables were assessed by the Spearman rank correlation coefficient. The PNI was Z-Score normalized for Cox regression analysis. Comparison of renal survival among different groups was evaluated by the Kaplan–Meier method. Cox regression analysis was performed to discriminate the risk factors for ESRD. The prediction of PNI at different times for ESRD was evaluated with time-dependent receiver operating characteristic (td-ROC) curve. Area under the receiver operating curve (AUC) was also used to establish the discrimination ability of different models. Integrated discrimination improvement (IDI) and net reclassification improvement (NRI) were calculated using logistic regression method to analyze the added prognostic value of PNI [19]. A two-way *p* < 0.05 was considered significant.

## 3. Results

### 3.1. Baseline Features of the Patients

A total of 321 individuals were included in this study (Figure 1). The demographic and clinical data of all the patients are shown in Table 1. The median PNI was 43.4 (36.53–49.78) in all the patients. The mean age was 51.64 ± 8.83 years old and 70.7% were male. The median DM duration was 96 (36–132) months. A total of 36.4% of the patients were early onset of T2DM, 52.5% were smokers, and 86.6% of patients had hypertension. The median of eGFR and proteinuria were 57.72 (41–86.02) mL/min/1.73 m^2^ and 3.81 (1.79–7.09) g/day, respectively. Among them, 69.47% of the subjects used RAS inhibitors.

### 3.2. Associations of PNI with Baseline Clinical Characteristics

We divided all the patients into the following three groups based on PNI: tertile 1 (<38.78), tertile 2 (38.78–47.12), and tertile 3 (≥47.12). In comparison with individuals with lower PNI, patients with higher PNI were more likely to have a lower incidence of hypertension, anemia, hypoalbuminemia, and RASI use; to have lower levels of proteinuria, serum creatinine, blood urea nitrogen, total cholesterol, LDL-C, HDL-C; to have higher levels of eGFR and uric acid, serum albumin, lymphocyte count. There was no difference in age, gender, DM duration, early onset of T2DM, smoking, body mass index, and HbA1c among individuals in different PNI tertiles.

### 3.3. Correlation of PNI with Clinical and Pathological Characteristics

**Figure 2:**
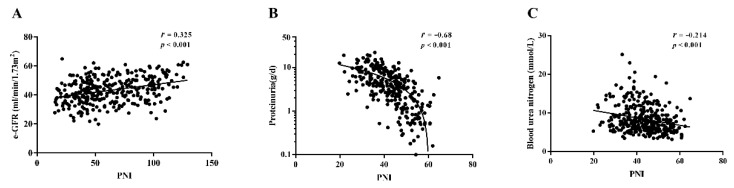
Correlations of prognostic nutritional index (PNI) with (**A**) eGFR, (**B**) urine protein, and (**C**) blood urea nitrogen among patients with diabetic nephropathy.

The correlation analysis demonstrated that the PNI level was positively related with eGFR (r = 0.325, *p* < 0.001), and negatively correlated with proteinuria (r = −0.68, *p* < 0.001) and blood urea nitrogen (r = −0.214, *p* < 0.001) in terms of clinical characteristics (Figure 2). Furthermore, there was a significant negative correlation between PNI and glomerular lesions (r = −0.412, *p* < 0.001), interstitial fibrosis and tubular atrophy (IFTA, r = −0.282, *p* < 0.001), interstitial inflammation (r = −0.271, *p* < 0.001) and arteriolar hyalinosis (r = −0.156, *p* = 0.005) in terms of renal pathological changes (Table 2).

### 3.4. PNI and ESRD in Patients with DN 

**Figure 3:**
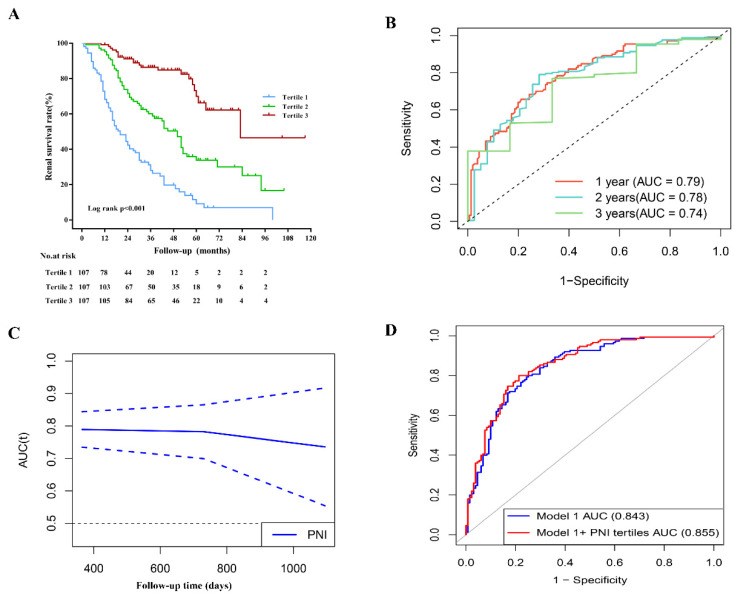
The prediction of PNI for ESRD in DN patients. (**A**) Kaplan–Meier curves of renal survival rate in patients with different PNI levels. (**B**,**C**) The prediction of PNI at different times for ESRD evaluated with time-dependent receiver operating characteristic (td-ROC) curve. (**D**) The prediction of models for ESRD evaluated with ROC curve.

During the median follow-up of 30 months (16–50 months), 164 of 321 (51.09%) patients progressed to ESRD. Comparing patients with lower PNI, those with higher PNI were likely to have a lower incidence of ESRD, as shown in Table 1. Kaplan–Meier analysis indicated that patients with higher PNI at baseline had a significantly lower risk for progression to ESRD (*p* < 0.001, Figure 3A) than patients with DN. Univariable Cox regression analysis suggested that per-SD increment in PNI was associated with decreased risk of ESRD (HR 0.445, 95% CI 0.38–0.522, *p* < 0.001), and higher tertiles were also predicted to lower the risk of ESRD (HR 0.368, 95% CI 0.296–0.456, *p* < 0.001), as shown in Table 3. In addition, early onset of T2DM, eGFR, proteinuria, anemia, hypoalbuminemia, glomerular lesion, IFTA, and RASI use were also risk factors for progression to renal failure (Supplement Table S1). After adjustment for baseline age, gender, hypertension, smoking, DM duration, early onset of T2DM, e-GFR, proteinuria, hypoalbuminemia, anemia, glomerular lesion, IFTA, and RASI use, per-SD increment in PNI was still associated with a lower incidence of ESRD (HR 0.705, 95% CI 0.523–0.952, *p* = 0.023). While hypoalbuminemia and anemia were not risk factors for ESRD after multivariate adjustment (Supplement Table S1). 

**Figure 4:**
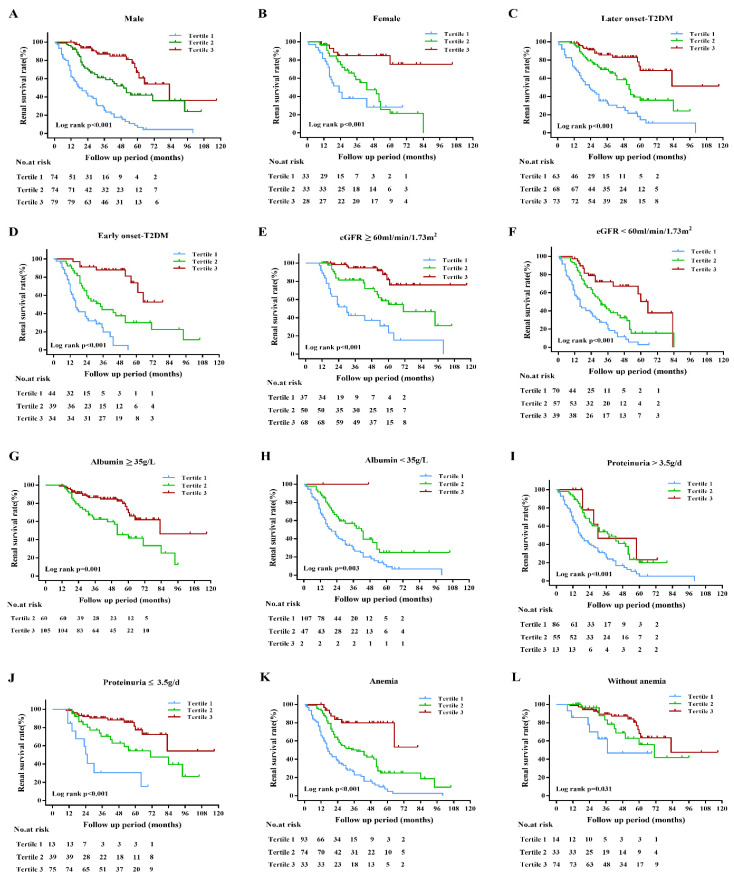
Kaplan–Meier curves of subgroup analysis in DN patients with different types of clinical manifestations. (**A**) The renal survival rate of male patients with different PNI tertiles. (**B**) The renal survival rate of female patients with different PNI tertiles. (**C**) The renal survival rate of DN patients with later-onset T2DM in different PNI tertiles. (**D**) The renal survival rate of DN patients with early onset T2DM in different PNI tertiles. (**E**,**F**) The renal survival rate of DN patients with eGFR ≥ 60 mL/min/1.73 m^2^ or <60 mL/min/1.73 m^2^ in different PNI tertiles. (**G**,**H**) The renal survival rate of DN patients with serum albumin ≥ 35 g/L or <35 g/L in different PNI tertiles. (**I**,**J**) The renal survival rate of DN patients with proteinuria > 3.5 g/day or ≤3.5 g/day in different PNI tertiles. (**K**,**L**) The renal survival rate of DN patients with or without anemia in different PNI tertiles.

We next investigated the association of PNI with ESRD in subgroups of patients with different clinical characteristics. The results demonstrated that higher PNI was still significantly associated with a reduced risk of ESRD in males and females (Figure 4A,B), in patients with or without the early onset of T2DM (Figure 4C,D), in patients with eGFR < 60 mL/min/1.73 m^2^ or not (Figure 4E,F), in patients with or without hypoalbuminemia (Figure 4G,H), in patients with proteinuria > 3.5 g/day or not (Figure 4I,J) and in patients with or without anemia (Figure 4K,L).

### 3.5. PNI and the Prediction of Incident ESRD

For the prediction of ESRD, the area under curves (td-AUC) evaluated with time-dependent receiver operating characteristics were 0.79 at 1 year, 0.78 at 2 years, and 0.74 at 3 years, respectively (Figure 3B,C). The AUC for prediction model 1 (including baseline age, gender, hypertension, smoking, DM duration, early onset of T2DM, e-GFR, proteinuria, hypoalbuminemia, and anemia) and prediction model 1 + PNI tertiles for progression to ESRD were 0.843(95% CI, 0.797–0.890) and 0.855 (95% CI, 0.811–0.900), respectively. Compared with those in model 1, the IDI (*p* = 0.011) and NRI (*p* < 0.001) in model 1 + PNI were significantly higher (Table 4 and Figure 3D).

## 4. Discussion

As far as we know, this was the first cohort research to investigate whether the PNI was associated with DN progression. In this study, PNI was evidenced to be an independent protective factor for kidney disease progression to ESRD in individuals with biopsy-confirmed DN and T2DM, even after adjustment for serum albumin and anemia. We observed that baseline PNI was positively related to eGFR, and negatively associated with proteinuria, glomerular injury, and IFTA. Our previous study suggested that baseline anemia and serum albumin were independent risk factors for incident ESRD in DN patients [20,21]. Additionally, several studies have shown that protein malnutrition is associated with CKD and ESRD [22]. These findings suggested that nutrition status may be a latent predictor of kidney disease progression.

To more accurately examine the prediction of PNI to ESRD, we performed different subgroup analyses. Grouped by gender, individuals with higher PNI tended to have higher survival rates compared with those with lower PNI in both males and females. In the subgroups of patients with different ages of T2DM diagnosis, patients with higher PNI had a lower risk of progression to ESRD in both ages < 40 y and age ≥ 40 y of T2DM diagnosis. Similarly, grouped based on the clinical features, PNI still was related to renal outcomes in patients with or without eGFR decline, even in the patients with mass proteinuria. We also found that the addition of PNI tertiles significantly enhanced the prediction power of DN developing to ESRD. The inclusion of PNI into model 1 based on traditional predictors improved the discrimination of model 1 for adverse renal outcomes with higher AUC, IDI, and NRI. These findings demonstrated that PNI might be an effective predictor for DN progression in T2DM patients. Undoubtedly, this conclusion awaits further large sample studies to confirm.

Interestingly, anemia and hypoalbuminemia have been evidenced to be associated with poor outcomes in diabetes with CKD [20,23]. However, in this study, PNI was still related to disease progression, while anemia and hypoalbuminemia were not. Furthermore, in the subgroups of patients without anemia or hypoalbuminemia, the PNI still predicted the risk of ESRD in DN. In line with these findings, we presumed that the PNI may be a better predictor than serum albumin, inflammation index, or lymphocyte count to predict the ESRD in DN patients, most likely because the PNI is a more comprehensive marker that reflects nutrition, immune and inflammation [24], all of which are closely related with DN. In addition, DN is a chronic inflammatory condition that may contribute to immune disorder and protein malnutrition, which in turn could exacerbate inflammation and create a vicious cycle to accelerate disease progression [10,22,25]. However, the hypothesis remains to be confirmed.

In particular, several studies have shown that the PNI was associated with kidney diseases. Hu et al. [24] and Dong et al. [26] revealed that PNI could predict the incident acute kidney injury (AKI) or mortality in patients with severe cardiac diseases. Zhang et al. [27] found that patients with a lower PNI had a more significant eGFR decline than those with higher PNI in children with CKD during follow-up. Additionally, as mentioned above, the PNI was also significantly related with mortality in elderly CKD patients [15]. These studies indicate that PNI may be very important for incident AKI and CKD prognosis.

The dietary protein intake may affect the PNI level and whether the DN patients should have a relatively low protein diet (LPD) is still controversial. In the ADA guidelines for the nutrition management of non-dialysis CKD in diabetic patients, a restricted dietary protein intake (DPI) of 0.8 g/kg/day, which is less than the average consumption level of Americans (1.2–1.4 g/kg/day) [28], is recommended to slow the renal function decline, but less than the level is not allowed [29]. Moreover, in the KDOQI guideline for nutrition recommendation in non-dialysis CKD with diabetes, it is appropriate to have an LPD of 0.6–0.8 g/kg/day to preserve a balanced nutritional state [30]. However, a recent study based on the DIALECT cohort suggested that high-normal dietary protein intake (>163 g/day) did not deteriorate renal function decline in T2DM patients [31], but a DPI < 92 g/day contributed to the renal function deterioration. Therefore, considering the influence of dietary protein intake on the PNI, whether the T2DM patients with kidney disease should have a low protein diet awaits more studies to clarify, and the management of individual precision nutrition will be the direction of future efforts.

Leukocytes in the peripheral blood have been evidenced to be involved in vascular endothelial cell injury in diabetes, and contribute to the development of atherosclerosis, cardiovascular disease, and kidney damage [32,33,34]. Our previous study demonstrated that the neutrophil-to-lymphocyte (NLR) was significantly associated with eGFR decline and histologic lesions in DN patients [35]. Furthermore, in this study, the lymphocyte count was also related to the risk of ESRD in univariate Cox analysis (HR 0.543, 95% CI 0.393–0.749, *p* < 0.001), but not emerging as an independent predictor in multivariate analysis. Relatively little research has been conducted on the relationship between absolute lymphocyte count and prognosis. The limited evidence [34,36,37,38] suggested that it is related to chronic inflammation markers, such as C-reactive protein, and predicted cardiovascular events and all-cause mortality in patients with or without diabetes. Cardoso et al. [34] also found the lymphocyte count did not involve in the development of microvascular complications, such as retinopathy outcome and renal function decline, in T2DM individuals, which was similar to ours. 

Of course, there are some limitations to our study. First, this research was conducted in a single center in Chinese patients with biopsy-proven DN, and it may therefore not be appropriate to generalize to other races and diabetic kidney disease diagnosed on the basis of clinical presentations. Second, our sample size is limited, and the patients included had a relatively short follow-up time. Third, some disrupting factors, such as dietary factors and the use of α ketoacid, were not considered. 

In summary, our findings afforded new evidence that PNI was a better predictor of the risks of DN progression than anemia and hypoalbuminemia in patients with T2DM. Furthermore, its addition to traditional risk factors, such as hypertension, smoking, proteinuria, eGFR, anemia, and hypoalbuminemia could improve the predictive ability for DN progression to renal failure in T2DM patients. Therefore, the utilization of PNI in estimating immunonutrition status condition might be very important in the comprehensive management of DN patients.

## Figures and Tables

**Table 1:** Clinical features of the patients stratified across the tertiles of PNI.

Valuable	All (*n* = 321)	Tertile 1 (*n* = 107) <38.78	Tertile 2 (*n* = 107) 38.78–47.12	Tertile 3 (*n* = 107) ≥47.12	*p* Value
PNI	43.4 (36.53–49.78)	34.05 (30.55–36.55)	43.4 (41.3–45.35)	52.7 (49.75–55.5)	<0.001
Age (years)	51.64 ± 8.83	51.83 ± 9.08	51.92 ± 7.92	51.18 ± 9.48	0.8
Gender (male, %)	227 (70.7)	74 (69.2)	74 (69.2)	79 (73.8)	0.687
DM duration (months)	96 (36–132)	96 (55–156)	120 (36–168)	96 (45–132)	0.474
Early onset of T2DM (%)	117 (36.4)	44 (41.1)	39 (36.4)	34 (31.8)	0.365
Smoking (%)	168 (52.5)	57 (53.3)	50 (47.2)	61 (57)	0.349
Body mass index (kg/m^2^)	25.65 (23.13–27.61)	24.61 (22.3–28.34)	25.781 (23.14–28.04)	25.93 (24.24–27.68)	0.372
MAP (mmHg)	105.59 ± 15.07	108.18 ± 15.4	105.00 ± 15.29	103.57 ± 14.26	0.072
Hypertension (%)	278 (86.6)	99 (92.5)	95 (88.8)	84 (78.5)	0.008
Initial proteinuria (g/day)	3.81 (1.79–7.09)	8.64 (4.35–10.5)	3.68 (2.58–6.82)	1.20 (0.54–2.58)	<0.001
e-GFR (ml/min/1.73 m^2^)	57.72 (41–86.02)	45.63 (35.80–67.3)	59.79 (43.8–78.67)	73.44 (46.11–99.55)	<0.001
Serum creatinine (μmol/L)	115 (80–154.5)	145 (106–167.5)	113 (87–137.6)	89 (73–140)	<0.001
BUN (mmol/L)	7.6 (5.9–10.13)	7.9 (6.83–13.29)	7.6 (5.9–9.9)	6.8 (5.18–8.98)	<0.001
Uric acid (μmol/L)	388.14 ± 79.98	372.23 ± 74.24	383.08 ± 78.70	409.1 ± 82.95	0.002
FBS (mmol/L)	7.15 (5.58–9.64)	8.13 (6.52–11.34)	7.37 (5.15–9.43)	6.98 (6.01–8.82)	0.18
HbA1c (%)	7.65 ± 1.87	7.7 ± 2.15	7.62 ± 1.82	7.65 ± 1.64	0.959
Triglyceride (mmol/L)	1.73 (1.27–2.33)	1.53 (1.22–2.18)	1.81 (1.31–2.31)	1.81 (1.25–2.66)	0.474
Total cholesterol (mmol/L)	4.95 (4.19–5.89)	5.26 (4.62–6.56)	5.13 (4.49–6.16)	4.16 (3.47–5.15)	<0.001
LDL-C (mmol/L)	2.83 (2.17–3.64)	2.95 (2.58–3.76)	3.03 (2.47–3.59)	2.21 (1.64–3.03)	<0.001
HDL-C (mmol/L)	1.21 (1.01–1.54)	1.35 (1.11–1.72)	1.15 (0.93–1.44)	1.14 (0.96–1.32)	<0.001
Anemia (%)	200 (62.3)	93 (86.9)	74 (69.2)	33 (30.8)	<0.001
Albumin (g/L)	35.2 (28.5–40.75)	28.2 (26.1–29.9)	35.4 (33.9–37.4)	42.9 (40.55–45.7)	<0.001
Hypoalbuminemia (%)	156 (48.6)	107 (100%)	47 (43.9)	2 (1.9)	<0.001
White blood cell (10^9^/L)	6.56 ± 1.67	6.35 ± 1.78	6.32 ± 1.62	7.01 ± 1.51	0.003
Lymphocyte (10^9^/L)	1.61 (1.3–2.03)	1.39 (1.17–1.74)	1.57 (1.29–1.94)	1.88 (1.54–2.28)	<0.001
RASI use (%)	223 (69.47)	83 (77.57)	82 (76.64)	58 (54.21)	<0.001
Insulin use (%)	256 (79.75)	84 (78.5)	87 (81.31)	85 (79.44)	0.874
Progressed to ESRD (%)	164 (51.09)	84 (78.5)	58 (54.21)	22 (20.56)	<0.001

Note: MAP, mean arterial pressure; BUN, blood urea nitrogen; e-GFR, estimated glomerular filtration rate; FBS, fasting blood sugar; LDL-C, low-density lipoprotein cholesterol; HDL-C, high-density lipoprotein cholesterol. RASI, renin–angiotensin system inhibitor. ESRD, end-stage renal disease. Data are presented as the mean ± standard, the median with (interquartile range) or counts and percentages.

**Table 2:** Pathologic features of patients with different PNI tertiles.

Pathological Lesions	All	Tertile 1	Tertile 2	Tertile 3	*p* #	r *	*p* *
Glomerular class					<0.001	−0.412	<0.001
I	16 (5)	0 (0)	1 (0.9)	15 (14)			
II a	68 (21.2)	8 (7.5)	20 (18.7)	40 (37.4)			
II b	42 (13.1)	12 (11.2)	13 (12.1)	17 (15.9)			
III	155 (48.3)	74 (69.2)	55 (51.4)	26 (24.3)			
IV	40 (12.5)	13 (12.1)	18 (16.8)	9 (8.4)			
IFTA					<0.001	−0.282	<0.001
0	9 (2.8)	1 (0.9)	1 (0.9)	7 (6.5)			
1	148 (46.1)	38 (35.5)	44 (41.1)	66 (61.7)			
2	134 (41.7)	53 (49.5)	51 (47.7)	30 (28)			
3	30 (9.3)	15 (14)	11 (10.3)	4 (3.7)			
Interstitial inflammation					<0.001	−0.271	<0.001
0	19 (5.9)	1 (0.9)	3 (2.8)	15 (14)			
1	234 (72.9)	74 (69.2)	80 (74.8)	80 (74.8)			
2	68 (21.2)	32 (29.9)	24 (22.4)	12 (11.2)			
Arteriolar hyalinosis					0.03	−0.156	0.005
0	32 (10)	7 (6.5)	7 (6.5)	18 (16.8)			
1	176 (54.8)	57 (53.3)	59 (55.1)	60 (56.1)			
2	113 (35.2)	43 (40.2)	41 (38.3)	29 (27.1)			

IFTA, interstitial fibrosis and tubular atrophy. # *p* value for the chi-squared test. * r: correlation coefficient analyzed using the Spearman test. * *p* value for the Spearman test.

**Table 3:** Associations between PNI level and renal outcomes.

	Per-SD Increment of PNI		PNI Tertiles		
	HR (95% CI)	*p* Value	HR (95% CI)	*p* Value	HR (95% CI)
Univariate	0.445 (0.38–0.522)	<0.001	0.368 (0.296–0.456)	<0.001	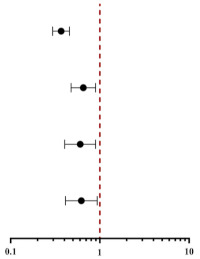
Model 1	0.694 (0.516–0.934)	0.016	0.642 (0.430–0.959)	0.030	
Model 2	0.702 (0.520–0.946)	0.020	0.645 (0.432–0.964)	0.033	
Model 3	0.705 (0.523–0.952)	0.023	0.649 (0.434–0.971)	0.036	

Note: PNI was analyzed as a continuous variable with hazard ratios (HRs) calculated per SD increment of PNI. **SD**, standard deviation; **CI**, confidence interval. **Model 1** adjusted for baseline age, gender, hypertension, smoking, DM duration, early onset of T2DM, e-GFR, proteinuria, hypoalbuminemia, and anemia. **Model 2** adjusted for covariates in model 1 plus renal pathological findings (the glomerular class and IFTA). **Model 3** adjusted for covariates in model 2 plus RASI use.

**Table 4:** Prediction of ESRD among DN patients.

Variables	Model 1	Model 1 + PNI Tertiles	*p* Value ^a^
AUC	0.843 (95% CI, 0.797–0.890)	0.855 (95% CI, 0.811–0.900)	0.10 ^b^
IDI	-	0.621 (95% CI, 0.390–0.844)	0.011
NRI	-	0.023 (95% CI, 0.005–0.040)	<0.001

Model 1 included baseline age, gender, hypertension, smoking, DM duration, early onset of T2DM, e-GFR, proteinuria, hypoalbuminemia and anemia; AUC, area under the curve; IDI, integrated discrimination improvement; 95% CI, confidence interval; NRI, net reclassification improvement. ^a^ *p* value (model 1 + PNI tertiles versus model 1. ^b^ DeLong’s test.

## Data Availability

The data presented in this study are available on request from the corresponding author. The data are not publicly available due to the protection of patient’s rights of privacy.

## Supplementary Materials

The following supporting information can be downloaded at: https://www.mdpi.com/article/10.3390/nu14173634/s1, Table S1: Cox regression analysis of risk factors for renal outcomes in DN patients.
