# Supplementary material for: Prognostic Nutritional Index as a Predictor of Diabetic Nephropathy Progression
Source: Nutrients. 2022 Sep 2;14(17):3634. doi: 10.3390/nu14173634 (PMC9460356; doi:10.3390/nu14173634)
Supplement: Supplementary file 1 [file nutrients-14-03634-s001.zip › nutrients-1842683-SI.pdf]

Supplementary -Table S1. Cox regression analysis of risk factors for renal outcomes in DN patients.

Variables	Unadjusted			Model 3		
	HR	95%CI	P value	HR	95%CI	P value
Age (yrs)	0.987	0.971-1.004	0.132	0.978	0.949-1.008	0.148
Gender (Male)	1.055	0.753-1.477	0.756	0.883	0.529-1.474	0.634
DM duration (months)	1	0.997-1.002	0.855	0.999	0.996-1.002	0.476
Early onset of T2DM	1.421	1.043-1.937	0.026	1.306	0.767-2.225	0.326
Smoking (yes or no)	1.117	0.821-1.520	0.481	1.510	0.946-2.409	0.084
Hypertension (yes or no)	1.588	0.97-2.601	0.066	0.907	0.492-1.670	0.754
e-GFR (ml/min/1.73 m2)	0.970	0.964-0.977	<0.001	0.975	0.965-0.984	<0.001
Proteinuria (g/d)	1.115	1.084-1.147	<0.001	1.023	0.979-1.069	0.306
Anemia (yes or no)	4.032	2.721-5.976	<0.001	1.468	0.872-2.472	0.149
Hypoalbuminemia (yes or no)	3.876	2.774-5.414	<0.001	1.608	0.926-2.790	0.091
Glomerular class	1.635	1.405-1.903	<0.001	1.148	0.919-1.434	0.223
IFTA	1.769	1.429-2.191	<0.001	0.834	0.625-1.115	0.220
RASI use	0.586	0.411-0.835	0.003	1.197	0.776-1.846	0.417
Per 1 SD increment of PNI	0.445	0.38-0.522	<0.001	0.705	0.523-0.952	0.023
